# Pharmacists’ knowledge, attitudes, beliefs, and barriers toward breast cancer health promotion: a cross-sectional study in the Palestinian territories

**DOI:** 10.1186/s12913-021-06458-5

**Published:** 2021-05-06

**Authors:** Ramzi Shawahna, Hiba Awawdeh

**Affiliations:** 1 Department of Physiology, Pharmacology and Toxicology, Faculty of Medicine and Health Sciences, An- Najah National University, P.O. Box 7, New Campus, Building: 19, Office: 1340, Nablus, Palestine; 2 An-Najah BioSciences Unit, Centre for Poisons Control, Chemical and Biological Analyses, An-Najah National University, Nablus, Palestine; 3 Faculty of Graduate Studies, An-Najah National University, Nablus, Palestine

**Keywords:** Awareness, Breast cancer, Pharmacists, Signs and symptoms, Screening, Health services

## Abstract

**Background:**

Among all cancers, breast cancer is the most prevalent cancer and the leading cause of mortality among women in developing countries including Palestine. Community pharmacists are trusted and easily accessible healthcare providers who could be engaged in breast cancer health promotion. This study was conducted with the aim of exploring knowledge, attitudes, beliefs, and barriers toward breast cancer health promotion among community pharmacists in the Palestinian territories.

**Methods:**

This study was conducted in a cross-sectional design using a questionnaire among community pharmacists. Knowledge of community pharmacists of breast cancer was tested using a 26-item knowledge test. Attitudes and beliefs of the community pharmacists with regard to breast cancer promotion were explored using 14 items. Barrier to breast cancer health promotion were explored using 9 items.

**Results:**

Data were collected from 200 community pharmacists. The median knowledge score was 69.2 % with and IQR of 15.2 %. Of the community pharmacists, 67.5 % scored 50 % and above in the knowledge test. Multivariate logistic regression showed that community pharmacists who were female in gender were more likely to score 50 % and above in the knowledge test compared to the community pharmacists who were male in gender (OR = 4.73, 95 % CI of 2.26–9.89). The community pharmacists had positive attitudes toward breast cancer health promotion. There was a significant moderate positive correlation between knowledge and attitudes scores (Spearman’s rho = 0.37, *p*-value < 0.001). Lack of reimbursement, lack of enough personnel, lack of time, and fear of offending the patients were the main barriers to breast cancer health promotion (percentage of agreement > 60.0 %).

**Conclusions:**

This study shed light on the role of community pharmacists in breast cancer health promotion. Pharmacists had good knowledge of breast cancer and positive attitudes toward promoting the health of patients with breast cancer. Further studies are still needed to determine how to integrate community pharmacists in the team of healthcare providers caring for patients with breast cancer.

**Supplementary Information:**

The online version contains supplementary material available at 10.1186/s12913-021-06458-5.

## Background

Cancer is a lethal disease that claims millions of human lives on yearly basis [1]. Among all cancers, breast cancer is the most prevalent cancer and the leading cause of mortality among women in developing countries [2–5]. It has been estimated that 1 in every 200 women would develop breast cancer under the age of 40 years [6]. According to the estimates of the International Agency for Research on Cancer, there were 19.3 million new cancer cases and 10.0 million cancer deaths in 2020 [7]. Despite the fact that breast cancer mortality rates have declined over the past 3 decades, the number of women living with breast cancer is expected to continue growing in some regions of the world [8–10]. In Palestine, breast cancer is the most prevalent type of cancer among women [11]. As in other low- and middle-income countries, little is known on the incidence and prevalence of breast cancer in Palestine [12]. According to the Palestinian Ministry of Health, the incidence of breast cancer was estimated at 33 per 100,000 in 2008 [13]. As breast cancer in Palestine is often diagnosed in late stages, breast cancer has been associated with a significant rate of mortality among Palestinian women [5, 11, 14]. According to recent statistics, breast cancer mortality accounts for 12 % of all cancer mortality among the Palestinians [13].

Breast self-examination and clinical breast examination can be combined for early detection of breast cancer. This combination was advocated by the international guidelines including those of the American Cancer Society [15]. It has been argued that early discovery of breast cancer improves survival rate [9, 16–19]. Early screening, detection, and treatment might save the breast and improve the chances of full recovery [16]. Despite the initiatives to screen for breast cancer, many patients show up for the first time to medical centers with advanced stages of breast cancer. This might indicate a need for increasing awareness of breast cancer among women as well as healthcare providers [14]. Lack of screening for breast cancer can be attributed to costs, lack of awareness of the importance of screening, unavailability of screening tools, and probably, cultural and social embarrassment to use the available screening methods [20–23]. After detection, many treatment modalities are available which usually take long time, need adherence, and continuous counseling. It has been argued that without adequate counseling and strict adherence, many of these therapeutic modalities might fail [24].

Pharmacists are important healthcare providers in many healthcare systems around the world. In addition to their expertise in dispensing medications, pharmacists can counsel and educate patients on how to make the best out of their therapeutic modalities [25–27]. In modern healthcare systems, the role of pharmacist has grown beyond merely dispensing medications and, today, pharmacists play a significant role in provision of patient-centered direct care [28–30]. Pharmacists are one of the most accessible and trusted healthcare providers. Community pharmacies are well located within the communities, remain open for extended working hours, and provide free counseling and educational services to patients [25]. Because women represent a considerable percentage of the clientele visiting community pharmacies, pharmacists can play a greater role in increasing awareness of breast cancer, counsel women on the availability of tools to screen for breast cancer and educate patients on the right ways of using their anticancer therapies [31, 32]. Pharmacists can also screen for adherence to anticancer therapies, side effects, drug-drug, drug-food, and drug-herb interactions [33–36]. Tanaka et al. reported that counseling by pharmacists increased the quality of life and reduced the side effects associated with chemotherapy among patients with breast cancer [37]. A previous study showed that patients with breast cancer who received counseling and education with regard to their treatment and side effects by pharmacists had less anxiety and better psychological outcomes compared to patients who did not receive counseling and education [38]. In another study, pharmacists answered questions of patients with breast cancer after receiving the first dose of chemotherapy [39]. The study showed that the vast majority (94.7 %) of the patients were “very satisfied” or “satisfied” with the answers they received from the pharmacists.

To effectively engage in breast cancer health promotion, pharmacists should have adequate knowledge of issues related to breast cancer, positive attitudes, beliefs, and willingness to provide pharmaceutical services to patients with breast cancer [30, 40, 41]. Previous studies have identified considerable knowledge gaps among pharmacists with regard to breast cancer in different settings around the world [40, 42–47]. These knowledge gaps might have limited engagement of pharmacists in breast cancer health promotion [48].

In Palestine, little is known on the knowledge, attitudes, and beliefs of community pharmacists with regard to breast cancer. Similarly, little is known on the willingness to and degree of engagement of community pharmacists in promoting the health of patients with breast cancer and barriers to providing breast cancer health promotion to patients visiting the pharmacy.

This study was conducted among community pharmacists in the Palestinian territories to explore their knowledge, attitudes, beliefs, and barriers toward breast cancer health promotion. Another aim of this study was to investigate the association of sociodemographic and practice variables of the community pharmacists with their knowledge and attitudes toward breast cancer health promotion.

## Methods

### Study design

This study was conducted in a cross-sectional observational design among community pharmacists using a pre-validated questionnaire as the study tools. This study is being reported in adherence to the Strengthening the Reporting of Observational Studies in Epidemiology (STROBE) Statement for reporting cross-sectional studies. Adherence to STROBE statement is shown in Supplementary file 1.

### Recruitment, inclusion, and exclusion criteria

The study population was community pharmacists in the West Bank of Palestine. In the West Bank of Palestine, there were 1,062 community pharmacies distributed in the main cities and villages of each governorate [13]. The distribution of pharmacies in each governorate is shown in Supplementary file 2. In this study, we aimed to recruit at least 1 pharmacist from each community pharmacy.

Pharmacists were visited by the field researcher in their places of work. The design and objectives of the study were explained to the potential participants before obtaining their written consent to participate in the study. In case the community pharmacists did not have time at the first visit, they were requested to provide timing for another appointment so the field researcher could return at their convenience. In this study, pharmacists who were licensed to practice community pharmacy in Palestine and were willing to respond to items in a questionnaire were included. Pharmacy assistants, students and trainees who were not licensed at the time of the study were not included.

### The study tool

The questions included in the questionnaire that was used in this study were adopted from the literature [40, 42, 48–50]. The questionnaire included 4 sections. In the first section, the sociodemographic and practice variables of the community pharmacists like age, gender, marital status, academic degrees, place from where the academic degree was obtained, number of years in practice, whether the pharmacist had ever attended a structured or organized continuing educational program on breast cancer, approximate number of patients with breast cancer interacted with per month, the pharmacist’s perceived knowledge of breast cancer, average number of working hours per week, average number of patients interacted with per day, percentage of female patients visiting the pharmacy, number of pharmacists working in the pharmacy at any one shift, frequency of responding to patient inquiries related to breast cancer warning signs and symptoms and breast cancer early detection and screening tests, frequency of providing patients with advice or counseling on breast cancer screening and early detection, frequency of providing patients with breast cancer educational materials or self-assessment quizzes, and frequency of referring patients to special breast cancer screening programs organized by hospitals or cancer organizations were collected. For the frequency questions, “rarely” was defined as sporadically or once a week and “often” was defined as on daily or almost on daily basis. The second section contained a 26-item knowledge test relevant to prevalence, risk factors, signs and symptoms, screening methods, and treatment of breast cancer. The community pharmacists had to answer each item by selecting either true/false/I don’t know. The third section contained 14 items relevant to attitudes and beliefs of the pharmacists with regard to providing health promotion advice to patients on breast cancer. The community pharmacists had to respond to each statement by either disagree/neutral/agree. The fourth section contained 9 barriers to providing breast cancer health promotion to patients visiting the pharmacy. The community pharmacists had to rate each barrier by either disagree/neutral/agree.

### Face validity, pilot testing, stability of scores, and internal consistency of the study tool

The questionnaire used in this study was reviewed by 2 oncologists, 2 community pharmacists, 2 clinical pharmacists with experience in caring for patients with breast cancer. Items included in the questionnaire were rated for relevance on a scale of 1–5 (1 indicated that the item was not relevant at all and 5 indicated that the item was highly relevant) [51]. It was decided *a priori* that items rated as relevant and highly relevant by the reviewers will be included in the final questionnaire. Conflicting ratings were resolved by discussion and consensus.

The study tool was pilot tested before it was used to ensure comprehensibility and clarity. Community pharmacists (n = 20) were asked to complete the questionnaire and after a short time period (30 min to 1 h), the same community pharmacists were given another copy of the questionnaire to complete in a second round.

### Data analysis

The test-retest method was used to test the stability of scores over a short period of time. Scores of both rounds were correlated using Pearson’s correlation. It was decided *a priori* that a correlation coefficient of > 80 % would be acceptable to ensure stability of the scores [52]. The internal consistency of the items included in the questionnaire was tested using Cronbach’s alpha. The internal consistency would be ensured by a Cronbach’s alpha of > 70 % [53, 54].

The data collected in this study were entered into IBM SPSS v.21.0. A two-way mixed model was used to compute value of Cronbach’s alpha with their 95 % CI. The p-value was estimated using Hotelling’s T-Squared Test.

To create categories, variables like age, number of patients with breast cancer interacted with per month, number of working hours per week, and number of patients interacted with per day were split around the median. For the knowledge items, the community pharmacists were awarded 1 point for each correct answer, 0 point for each I don’t know answer, and 0.5 point was deducted as a penalty for each wrong answer [55, 56]. Currently, there is no consensus on the cut-off that indicates adequate knowledge in a certain domain. In this study, scoring ≥ 50 % in the knowledge test was used as in previous studies in which knowledge of pharmacists was tested [55, 57].

Scores were assessed for normality of distribution using Shapiro-Wilk test. As the distribution was not normal, data were expressed using medians and interquartile range (IQR). Correlations between variables in the categorical groups were investigated using Chi-square (χ^2^)/Fisher’s Exact Test and Spearman’s correlations. To identify predictors of good knowledge, multivariate logistic regression was conducted retaining variables that were significantly associated in the χ^2^/Fisher’s Exact Test and Spearman’s correlations [57, 58]. Enter method was used. Statistical significance was indicated by a p-value of < 0.05.

### Ethics approval and consent to participate

 This study was conducted in accordance with the principles in the Declaration of Helsinki and those followed at An-Najah National University. The protocol and ethics of this study were approved by the Institutional Review Board (IRB) of An-Najah National University (An-Najah National University IRB-Protocol # 26-Oct-17). The community pharmacists who took part in this study provided written informed consent before their participation.

## Results

### Stability of scores and internal consistencies

The pilot testing showed that the questionnaire had excellent stable scores over a short period of time as indicated by the Pearson’s correlation coefficients of > 90 % (95 % CI = 0.91–0.99, p-value < 0.001). Additionally, the items included in the questionnaire were internally consistent as indicated by an overall Cronbach’s alpha of 83.9 % (95 % CI = 81.4-85.9 %, p-value < 0.001). Cronbach’s alpha values were also computed for each individual domain separately. The values of Cronbach’s alpha were 82.3 % (95 % CI = 78.5-85.6 %, p-value < 0.001), 85.5 % (95 % CI = 82.4-88.3 %, p-value < 0.001), and 73.3 % (95 % CI = 70.2-76.3 %, p-value < 0.001) for the 26 knowledge items, 14 attitudes and beliefs items, and 15 barrier items, respectively.

### The community pharmacists who took part in the study

In this study, complete questionnaires were returned by 200 of the 300 invited pharmacists, giving a response rate of 66.7 %. Of the those, 133 (66.5 %) were female in gender, 158 (79.0 %) had a Bachelor of Science (BSc) degree in pharmacy, 114 (57.0 %) had a practical experience of more than 5 years, 180 (90.0 %) had not attended an educational program on breast cancer, 43 (21.5 %) interacted with 3 and more patients with breast cancer, 118 (59.0 %) perceived their knowledge of breast cancer as good to excellent, 120 (60.0 %) worked 40 and more hours per week, 80 (40.0 %) interacted with 50 and more patients per day, 59 (29.5 %) stated that they often responded to patient inquiries related to breast cancer warning signs and symptoms and breast cancer early detection and screening tests, 42 (21.0 %) stated that they often provided patients with breast cancer educational materials or self-assessment quizzes, and 41 (20.5 %) stated that they often referred patients to special breast cancer screening programs organized by hospitals or cancer organizations. The detailed sociodemographic and practice variables of the pharmacists who took part in the study are shown in Table 1.

**Table 1:** Sociodemographic and practice variables of the pharmacists who took part in the study (*n = 200*)

Variable	n	%
**Age (years)**		
< 30	100	50.0
≥ 30	100	50.0
**Gender**		
Male	67	33.5
Female	133	66.5
**Marital status**		
Never married	74	37.0
Married/divorced/widowed	126	63.0
**Education**		
BSc Pharmacy	158	79.0
Pharm.D	18	9.0
Postgraduate pharmacy education (MSc or PhD)	24	12.0
**Place from where the academic degree was obtained**		
Palestine	152	76.0
Other	48	24.0
**Experience (years)**		
< 5	86	43.0
5 to < 10	43	21.5
≥ 10	71	35.5
**Attended an educational program on breast cancer**		
No	180	90.0
Yes	20	10.0
**Approximate number of patients with breast cancer interacted with per month**		
< 3	157	78.5
≥ 3	43	21.5
**Perceived knowledge of breast cancer**		
Poor-Fair	82	41.0
Good-Excellent	118	59.0
**Average number of working hours per week**		
< 40	80	40.0
≥ 40	120	60.0
**Average number of patients interacted with per day**		
< 50	120	60.0
≥ 50	80	40.0
**Percentage (%) of female patients visiting the pharmacy**		
< 20	128	64.0
20 %-49	38	19.0
≥ 50	34	17.0
**Number of pharmacists working in the pharmacy at any one shift**		
1	96	48.0
2 or more	104	52.0
**Respond to patient inquiries related to breast cancer warning signs and symptoms and breast cancer early detection and screening tests**		
Rarely	141	70.5
Often	59	29.5
**Provide patients with advice or counseling on breast cancer screening and early detection**		
Rarely	142	71.0
Often	58	29.0
**Provide patients with breast cancer educational materials or self-assessment quizzes**		
Rarely	158	79.0
Often	42	21.0
**Refer patients to special breast cancer screening programs organized by hospitals or cancer organizations**		
Rarely	159	79.5
Often	41	20.5

*BSc* Bachelor of Science, *Pharm.D* Doctor of Pharmacy, *MSc* Master of Science, *PhD* Doctor of Philosophy

### Knowledge of community pharmacists with regard to breast cancer

In this study, 67.5 % of the pharmacists scored 50 % and above in the knowledge test. Pharmacists had good knowledge of prevalence of breast cancer, the risk factors associated with breast cancer, signs and symptoms of breast cancer, screening methods to detect breast cancer, and treatment options available for the management of breast cancer as indicated by the number of correctly answered questions. The median score was 55.8 % with and IQR of 21.2 %. In this study, 178 (89.0 %) were aware that breast cancer was the most common form of cancer among women and 156 (78.0 %) were aware that breast cancer should be a concern even for women younger than forty years old.

With regard to the risk factors, 159 (79.5 %) were aware that hormone replacement therapy was a risk factor associated with developing breast cancer, 68 (34.0 %) were aware that late onset menstrual period was not a risk factor of developing breast cancer, 145 (72.5 %) were aware that old age increase the risk of breast cancer, 129 (64.5 %) were aware that cigarette smoking was associated with breast cancer, 176 (88.0 %) were aware that family history was associated with development of breast cancer, 154 (77.0 %) were aware that using oral contraceptives did not reduce the risk of developing breast cancer, 63 (31.5 %) were aware that breast size was associated with developing breast cancer, and 180 (90.0 %) were aware that breastfeeding did not increase the risk of breast cancer.

With regard to signs and symptoms, 159 (79.5 %) were aware that nipple discharge could be a sing of breast cancer, 130 (65.0 %) were aware that painless breast lump under armpit could be a sing of breast cancer, 171 (85.5 %) were aware that change in the breast shape could be a sign of breast cancer, and 126 (63.0 %) were aware that pain in breast region, retraction, or dimpling were signs of breast cancer.

With regard to screening, 185 (92.5 %) were aware that appropriate early screening for breast cancer reduced breast cancer mortality, 184 (92.0 %) were aware that breast self-examination was one of the methods used to detect the presence of breast cancer, 44 (22.0 %) were aware that frequent breast self-examination could be needed to detect the presence of breast cancer, 100 (50.0 %) were aware that mammography was not painful, 137 (68.5 %) were aware that mammography was safe, and 90 (45.0 %) were aware that breast self-examination was recommended for women aged 35 and below and should be done once a month.

With regard to treatment, 99 (49.5 %) were aware that total mastectomy was not the surgical option of choice for patients diagnosed with early stage breast cancer, 119 (59.5 %) were aware that patients with invasive breast cancer that was estrogen receptor positive should receive adjuvant endocrine therapy, 153 (76.5 %) were aware that tamoxifen was the adjuvant endocrine therapy of choice for premenopausal patients with invasive breast cancer, 117 (58.5 %) were aware that treatment of breast cancer was a long and painful process, and 93 (46.5 %) were aware that treatment of breast cancer does not necessarily affect fertility.

Detailed responses of the pharmacists in this study are shown in Table 2.

**Table 2:** Responses of the community pharmacists on the 26-item knowledge test on the prevalence, risk factors, signs and symptoms, screening methods, and treatment of breast cancer

			Correct answer		Incorrect answer		Did not know	
#	Question	Correct answer	n	%	n	%	n	%
	**Prevalence of breast cancer**							
1	Breast cancer is the most common form of cancer among women.	T	178	89.0	8	4.0	14	7.0
2	Breast cancer should not be of concern for patients younger than forty years of age.	F	156	78.0	30	15.0	14	7.0
	**Risk factors**							
3	Use of hormone replacement therapy is one of the risk factors for developing breast cancer.	T	159	79.5	20	10.0	21	10.5
4	Late onset menstrual period is one of the risk factors for developing breast cancer.	F	68	34.0	58	29.0	74	37.0
5	Old age increase risk of breast cancer.	T	145	72.5	34	17.0	37	18.5
6	There is no relationship between cigarette smoking and breast cancer.	F	129	64.5	34	17.0	12	6.0
7	Family history of breast cancer is a risk of breast cancer.	T	176	88.0	12	6.0	12	6.0
8	Use of oral contraceptive decrease risk of breast cancer.	F	154	77.0	27	13.5	19	9.5
9	There is no relationship between size of breast and risk of breast cancer.	F	63	31.5	102	51.0	35	17.5
10	Breastfeeding increase risk of breast cancer.	F	180	90.0	8	4.0	12	6.0
	**Signs and symptoms**							
11	Nipple discharge can be a warning sign of breast cancer.	T	159	79.5	19	9.5	22	11.0
12	Painless breast lump under armpit is not sign of breast cancer.	F	130	65.0	32	16.0	38	19.0
13	Change in breast shape is a sign of breast cancer.	T	171	85.5	15	7.5	14	7.0
14	Pain in breast region and dimpling are signs of breast cancer.	T	126	63.0	52	26.0	22	11.0
	**Screening methods**							
15	Appropriate early screening for breast cancer reduces breast cancer mortality.	T	185	92.5	5	2.5	10	5.0
16	Breast self-examination is one of the methods that are used to detect the presence of breast cancer.	T	184	92.0	7	3.5	9	4.5
17	To detect the presence of breast cancer, women over the age of twenty and under the age of forty should do a breast self-examination at least once per year.	F	44	22.0	136	68.0	20	10.0
18	To detect the presence of breast cancer, women aged forty years and above should do a monthly breast self-examination, an annual clinical breast examination and a biannual mammogram.	F	17	8.5	161	80.5	23	11.5
19	Mammography is painful.	F	100	50.0	49	24.5	51	25.5
20	Mammography is safe.	T	137	68.5	17	8.5	46	23.0
21	Breast self-examination is recommended for women aged 35 and below and should be done once a month.	T	90	45.0	59	29.5	51	25.5
	**Treatment**							
22	Total mastectomy is the surgical option of choice for patients diagnosed with early-stage breast cancer.	F	99	49.5	73	36.5	28	14.0
23	Patients with invasive breast cancer that is estrogen receptor positive should receive adjuvant endocrine therapy.	T	119	59.5	17	8.5	64	32.0
24	Tamoxifen is the adjuvant endocrine therapy of choice for premenopausal patients with invasive breast cancer.	T	153	76.5	51	25.5	32	16.0
25	The treatment of breast cancer is a long and painful process.	T	117	58.5	35	17.5	11	5.5
26	Treatment of breast cancer affects fertility.	F	93	46.5	35	17.5	72	36.0

*T* true, *F* false

### Attitudes and beliefs of pharmacists with regard to providing advice to patients on breast cancer

With regard to attitudes and beliefs of the community pharmacists on providing advice to patients with breast cancer, the pharmacists were generally positive as indicated by the number of pharmacists who agreed to the attitudes and beliefs statements. There was significant moderate positive correlation between the knowledge scores and attitudes scores (Spearman’s rho = 0.37, p-value < 0.001).

In this study, only 18 (9.0) disagreed with the statement regarding being involved in breast cancer health promotion activities, 19 (9.5 %) disagreed with the statement on the importance of integrating breast cancer health promotion into their daily practice, 13 (6.5 %) disagreed with the statement on feeling confident and prepared to provide breast cancer health promotion, 8 (4.0 %) disagreed with the statement on discussing breast cancer awareness with female patients in the pharmacy was beneficial and could save lives, 39 (19.5 %) disagreed with the statement on providing breast cancer counseling to female patients in the pharmacy was their responsibility, 14 (7.0 %) disagreed with the statement on distributing breast cancer educational materials was important, 7 (3.5 %) disagreed with the statement on the importance of discussing breast cancer with female patients to encourage breast cancer early screening and detection, 27 (13.5 %) disagreed with the statement on the evidence that pharmacist could influence patients to adopt breast cancer screening practices, 29 (14.5 %) disagreed with the statement on the importance of inviting healthcare professionals to provide breast cancer education to female patients in the pharmacy, 9 (4.5 %) disagreed with the statement on improving professional state and increasing professional satisfaction through providing breast cancer counseling, 11 (5.5 %) disagreed with the statement on providing breast cancer counseling was an effective use of their time, 12 (6.0 %) disagreed with the statement on the likely to providing breast cancer health promotion to female patients in case they had access to patient education materials related to breast cancer, 29 (14.5 %) disagreed with the statement on the willingness of the patients to counsel them on breast cancer screening and early detection, and 21 (10.5 %) disagreed with the statement that patients would appreciate their efforts to counsel them on breast cancer. Details of the responses are shown in Table 3.

**Table 3:** Responses of the pharmacists on the belief statements with regard to providing advice to patients on breast cancer

		Disagree		Neutral		Agree	
**#**	**Statement**	**n**	**%**	**n**	**%**	**n**	**%**
1	I should be involved in breast cancer health promotion activities in the pharmacy	18	9.0	35	17.5	147	73.5
2	Integrating breast cancer health promotion into my daily practice as a community pharmacist is important	19	9.5	41	20.5	140	70.0
3	I feel confident and prepared to provide breast cancer health promotion	13	6.5	40	20.0	147	73.5
4	Discussing breast cancer awareness with my female patients in the pharmacy is beneficial and can save their lives	8	4.0	30	15.0	162	81.0
5	Providing breast cancer counseling to my female patients in the pharmacy is my responsibility as a pharmacist	39	19.5	41	20.5	119	59.5
6	Distributing breast cancer educational materials is important in the pharmacy	14	7.0	40	20.0	146	73.0
7	It is important to discuss breast cancer with my female patients to encourage breast cancer early screening and detection	7	3.5	42	21.0	151	75.5
8	There is enough evidence to suggest that the pharmacist can influence patients to adopt breast cancer screening practices	27	13.5	56	28.0	117	58.5
9	Inviting healthcare professionals to provide breast cancer education to the female patients in the pharmacy is important	29	14.5	56	28.0	115	57.5
10	Providing breast cancer counseling to my patients can improve my professional state and increase my professional satisfaction	9	4.5	39	19.5	152	76.0
11	Providing breast cancer counseling is an effective use of my time	11	5.5	60	30.0	129	64.5
12	If I have access to patient education materials related to breast cancer, I am more likely to provide breast cancer health promotion to my female patients	12	6.0	45	22.5	143	71.5
13	Patients would like me as a pharmacist to counsel them on breast cancer screening and early detection	29	14.5	58	29.0	113	56.5
14	Patients appreciate my effort as a pharmacist to counsel them on breast cancer	21	10.5	59	29.5	120	60.0

### Barriers to providing breast cancer health promotion to patients visiting the pharmacy

In this study, 86 (43.0 %) agreed that patients not asking for breast cancer health promotion, 107 (53.5 %) agreed that patients not appreciating the pharmacist’s role as a breast cancer health promoter, 132 (66.0 %) patients feeling offended by breast cancer counseling offers, 130 (65.0 %) absence of reimbursements for providing breast cancer health promotion, 136 (68.0 %) agreed that absence of enough personnel in the pharmacy to provide breast cancer health promotion to patients visiting the pharmacy, 122 (61.0 %) agreed that not having enough time to provide breast cancer health promotion to patients visiting the pharmacy, 124 (62.0 %) agreed that not having enough space to provide breast cancer health promotion to patients visiting the pharmacy, 95 (47.5 %) agreed that not having access to breast cancer educational materials, and 83 (41.5 %) agreed that absence of support to the pharmacist’s role as a breast cancer health promoter by the pharmacy manager were barriers to providing breast cancer health promotion to patients visiting the pharmacy. Detailed responses of the pharmacists are provided in Table 4.

**Table 4:** Barrier to providing breast cancer health promotion to patients visiting the pharmacy

		Disagree		Neutral		Agree	
**#**	**Barrier**	**n**	**%**	**n**	**%**	**n**	**%**
1	Patients do not ask for breast cancer health promotion	33	16.5	81	40.5	86	43.0
2	Patients do not appreciate the pharmacist’s role as a breast cancer health promoter	41	20.5	52	26.0	107	53.5
3	Patients will be offended if I offer them breast cancer counseling	42	21.0	26	13.0	132	66.0
4	I am not reimbursed for providing breast cancer health promotion	46	23.0	24	12.0	130	65.0
5	Availability of enough personnel in the pharmacy to provide breast cancer health promotion to patients visiting the pharmacy	34	17.0	30	15.0	136	68.0
6	Having enough time to provide breast cancer health promotion to patients visiting the pharmacy	18	9.0	60	30.0	122	61.0
7	Having enough space to provide breast cancer health promotion to patients visiting the pharmacy	27	13.5	49	24.5	124	62.0
8	Have access to breast cancer educational materials	42	21.0	63	31.5	95	47.5
9	The pharmacy manager does not support my role as a breast cancer health promoter	93	46.5	24	12.0	83	41.5

### Associations between sociodemographic and practice variables of the pharmacists and their knowledge of breast cancer

Chi-square and Spearman’s correlations have shown that there was significant association between some sociodemographic and practice variables of the pharmacists and their knowledge of breast cancer as indicated by scoring more than 50 % in the knowledge test. Details of these associations are shown in Table 5.

**Table 5:** Associations between sociodemographic and practice variables of the pharmacists and scoring 50 % or more in the knowledge test

			Knowledge score							
			**< 50 %**		**≥ 50 %**					
**Variable**	**n**	**%**	**n**	**%**	**n**	**%**	**χ**^**2**^**/Fisher’s Exact Test**	***p*-value**	**Correlation**	***p*-value**
**Age (years)**										
< 30	100	50.0	23	11.5	77	38.5	8.23	0.006	-0.20	0.004
≥ 30	100	50.0	42	21.0	58	29.0				
**Gender**										
Male	67	33.5	38	19.0	29	14.5	26.93	< 0.001	0.37	< 0.001
Female	133	66.5	27	13.5	106	53.0				
**Marital status**										
Never married	74	37.0	18	9.0	56	28.0	3.58	0.063	-0.13	0.059
Married/divorced/widowed	126	63.0	47	23.5	79	39.5				
**Education**										
BSc Pharmacy	158	79.0	58	29.0	100	50.0	6.08	0.045	0.17	0.014
Pharm.D	18	9.0	3	1.5	15	7.5				
Postgraduate pharmacy education (MSc or PhD)	24	12.0	4	2.0	20	10.0				
**Place from where the academic degree was obtained**										
Palestine	152	76.0	40	20.0	112	56.0	11.04	0.001	-0.23	0.001
Other	48	24.0	25	12.5	23	11.5				
**Experience (years)**										
< 5	86	43.0	23	11.5	63	31.5	6.27	0.045	0.15	0.030
5 to < 10	43	21.5	11	5.5	32	16.0				
≥ 10	71	35.5	31	15.5	40	20.0				
**Attended an educational program on breast cancer**										
No	180	90.0	61	30.5	119	59.5	1.58	0.314	0.09	0.210
Yes	20	10.0	4	2.0	16	8.0				
**Approximate number of patients with breast cancer interacted with per month**										
< 3	157	78.5	49	24.5	108	54.0	0.55	0.467	-0.05	0.459
≥ 3	43	21.5	16	8.0	27	13.5				
**Perceived knowledge of breast cancer**										
Poor-Fair	82	41.0	28	14.0	54	27.0	0.17	0.759	0.03	0.680
Good-Excellent	118	59.0	37	18.5	81	40.5				
**Average number of working hours per week**										
< 40	80	40.0	26	13.0	54	27.0	0.00	1.000	0.00	1.000
≥ 40	120	60.0	39	19.5	81	40.5				
**Average number of patients interacted with per day**										
< 50	120	60.0	39	19.5	81	40.5	0.00	1.000	0.00	1.000
≥ 50	80	40.0	26	13.0	54	27.0				
**Percentage of female patients visiting the pharmacy**										
< 20 %	128	64.0	39	19.5	89	44.5	1.08	0.592	0.05	0.494
20-49 %	38	19.0	15	7.5	23	11.5				
≥ 50 %	34	17.0	11	5.5	23	11.5				
**Number of pharmacists working in the pharmacy at any one shift**										
1	96	48.0	31	15.5	65	32.5	0.00	1.000	0.00	0.952
> 1	104	52.0	34	17.0	70	35.0				
**Respond to patient inquiries related to breast cancer warning signs and symptoms and breast cancer early detection and screening tests**										
Rarely	141	70.5	44	22.0	97	48.5	0.37	0.620	-0.04	0.548
Often	59	29.5	21	10.5	38	19.0				
**Provide patients with advice or counseling on breast cancer screening and early detection**										
Rarely	142	71.0	45	22.5	97	48.5	0.15	0.741	-0.03	0.704
Often	58	29.0	20	10.0	38	19.0				
**Provide patients with breast cancer educational materials or self-assessment quizzes**										
Rarely	158	79.0	50	25.0	108	54.0	0.25	0.711	-0.04	0.619
Often	42	21.0	15	7.5	27	13.5				
**Refer patients to special breast cancer screening programs organized by hospitals or cancer organizations**										
Rarely	159	79.5	52	26.0	107	53.5	0.01	1.000	0.01	0.904
Often	41	20.5	13	6.5	28	14.0				

*BSc* Bachelor of Science, *Pharm.D* Doctor of Pharmacy, *MSc* Master of Science, *PhD* Doctor of Philosophy

In this study, pharmacists who were younger than 30 years old (χ^2^/Fisher’s Exact Test = 8.23, *p-*value = 0.006, Spearman’s rho = -0.20, p-value = 0.004), female in gender (χ^2^/Fisher’s Exact Test = 26.93, *p*-value < 0.001, Spearman’s rho = 0.37, *p*-value < 0.001), had higher education (χ^2^/Fisher’s Exact Test = 6.08, p-value = 0.045, Spearman’s rho = 0.17, *p*-value = 0.014), obtained their pharmacy degree from a Palestinian university (χ^2^/Fisher’s Exact Test = 11.04, *p*-value = 0.001, Spearman’s rho = -0.23, *p*-value = 0.001), and those who had longer practical experience (χ^2^/Fisher’s Exact Test = 6.27, *p*-value = 0.045, Spearman’s rho = 0.15, *p*-value = 0.030) scored significantly higher than pharmacists who were 30 and more years old, male in gender, obtained their pharmacy degree from outside, and had shorter practical experience. On the other hand, other variables like marital status, attending an educational program on breast cancer, number of patients with breast cancer interacted with per month, perceived knowledge of breast cancer, number of working hours per week, number of patients interacted with per day, percentage of female patients visiting the pharmacy, number of pharmacists working in the pharmacy at any one shift, responding to patient inquiries related to breast cancer warning signs and symptoms and breast cancer early detection and screening tests, providing patients with advice or counseling on breast cancer screening and early detection, providing patients with breast cancer educational materials or self-assessment quizzes, and referring patients to special breast cancer screening programs organized by hospitals or cancer organizations were not significantly associated with scoring more than 50 % in the knowledge test.

### Predictors of good knowledge

To identify the sociodemographic and practice variables that predicted scoring 50 % or more in the knowledge test, multivariate logistic regression showed that being of female gender was the only significant predictor of scoring 50 % or more in the knowledge test. The multivariate logistic regression showed that female pharmacists were 4.73-fold (95 % CI of 2.26–9.89) more likely to score 50 % or more in the knowledge test compared to male pharmacists. Details of the associations are shown in Table 6.

**Table 6:** Multivariate regression analysis of association between sociodemographic and practice variables with scoring 50 % or more in the knowledge test

						95 % CI for OR	
**Variable**	**β**	**SE**	**Wald**	***p*-value**	**OR**	**Lower**	**Upper**
**Age (years)**							
< 30	0.86	0.61	1.97	0.160	2.36	0.71	7.80
≥ 30	Reference category						
**Gender**							
Male	Reference category						
Female	1.55	0.38	17.02	< 0.001	4.73	2.26	9.89
**Education**							
BSc Pharmacy	Reference category						
Pharm.D	1.34	0.70	3.68	0.055	3.82	0.97	15.03
Postgraduate pharmacy education (MSc or PhD)	0.42	0.89	0.22	0.640	1.52	0.26	8.70
**Place from where the academic degree was obtained**							
Palestine	0.35	0.44	0.65	0.421	1.42	0.60	3.35
Other	Reference category						
**Experience (years)**							
< 5	Reference category						
5 to < 10	-0.82	0.67	1.52	0.218	0.44	0.12	1.63
≥ 10	-0.15	0.52	0.08	0.782	0.87	0.31	2.42

*BSc* Bachelor of Science, *Pharm.D* Doctor of Pharmacy, *MSc* Master of Science, *PhD* Doctor of Philosophy, *OR* Odds ratio, *SE* standard error.

## Discussion

In this study, pharmacists’ knowledge, attitudes, beliefs, and barriers toward breast cancer health promotion were exposed. To the best of our knowledge, this study is the first to explore pharmacists’ knowledge, attitudes, beliefs, and barriers toward breast cancer health promotion in Palestine. The main findings of this study highlighted awareness and knowledge gaps in some areas relevant to breast cancer among community pharmacists. On the other hand, community pharmacists were generally positive with regard to their engagement in breast cancer health promotion. Lack of reimbursement, lack of enough personnel, lack of time, and fear of offending the patients were the main barriers limiting their role in breast cancer health promotion. Findings of this study could inform future decisions relevant to breast cancer health promotion as detection of breast cancer in early stages increases the chances of survival significantly [9, 16]. Reports from Palestine and other developing countries showed low levels of awareness on how to perform breast self-examination by women themselves [59–62]. Taken together, findings of this study highlighted the necessity of higher engagement of community pharmacists in breast cancer health promotion.

The study questionnaire used in this study was informed by those used in previous studies elsewhere [40, 42, 48–50]. The questionnaire was re-validated before it was used in this study [52–54]. The questionnaire used in this study showed stable scores over a short period of time and good internal consistency. This indicated that the questionnaire used in this study was reliable and internally consistent. The sampling strategy used in this study ensured inclusion of a representative sample of community pharmacists practicing in Palestine. The sample included pharmacists from both genders, different age groups, educational levels, places from where the pharmacy degree was obtained, interaction with patients with breast cancer, experience, and training levels. This diversity in the sample might have added rigor and validity to the results obtained in this study.

In this study, 67.5 % of the community pharmacists scored 50 % and above in the knowledge test. Of the pharmacists, 89.0 % correctly answered the question on the prevalence of breast cancer. This percentage was higher than that reported among pharmacists in Malaysia and Jordan [40, 42] and lower than that reported among pharmacists in Qatar [48]. Similarly, 78.0 % correctly answered that women under 40 years old should also be concerned about breast cancer. This percentage was higher than that reported among pharmacists in Qatar and Jordan [40, 48]. With regard to knowledge of risk factors, pharmacists in this study were knowledgeable of using hormone replacement therapy, older age, breast size, cigarette smoking, and family history. Pharmacists were also knowledgeable that using oral contraceptives and breastfeeding were not associated with development of breast cancer. It has been argued that adequate knowledge of prevalence and risk factors of breast cancer is a prerequisite for engaging community pharmacists in breast cancer health promotion activities. With regard to signs and symptoms, pharmacists in this study were generally knowledgeable of the signs and symptoms of breast cancer like nipple discharge, painless breast lump under armpit, change in breast shape, and pain in breast region. These percentages were generally higher than those reported among pharmacists in Jordan, Malaysia, and Qatar [40, 42, 48]. However, only 34.0 % of the community pharmacists who participated in this study could correctly identify that late onset of menstrual period was one of the risk factors of breast cancer. Community pharmacists should be knowledgeable of the signs and symptoms of breast cancer as they need to counsel/educate women on how to recognize these signs and symptoms. In many cases, community pharmacists might need to answer questions raised by women with regard to some signs that could be considered potential red flags that need referrals to specialty services. With regard to the screening methods, the community pharmacists in this study were highly knowledgeable of the potential of reducing breast cancer mortality through early screening, breast self-examination, and safety of mammography. It has been argued that community pharmacists can play a greater role in promoting screening and early detection of breast cancer. In this study, knowledge gaps were identified with regard to frequency of conducting breast self-examination, clinical breast examination, and mammography. Some of these knowledge gaps were reported in previous studies [42, 48]. Findings of this study might shed more light on the need to improve knowledge of community pharmacists with regard to how frequent these examinations need to be performed. With regard to treatment modalities, more than 50 % of the pharmacists were knowledgeable of using adjuvant endocrine therapy for patients with estrogen receptor positive invasive breast cancer, using tamoxifen as the adjuvant endocrine therapy of choice for premenopausal patients with invasive breast cancer, and the length of treatment. However, knowledge gaps were identified with regard to the possibility of preserving the breast in patients diagnosed with early-stage breast cancer and fertility in breast cancer treatment. Some of these knowledge gaps were previously reported among pharmacists in Qatar [48]. Pharmacists are experts in medications whose role is to help patients how to make the best out of their treatment options. Additionally, the role of pharmacists also extends to help other healthcare professionals like oncologists optimize therapy and minimize adverse effects. In this study, knowledge scores were significantly associated with age, gender, higher education, length of experience, and obtaining the pharmacy degree from a Palestinian university. Findings of this study were consistent with those reported in Jordan among community pharmacists [40]. When the multivariate logistic regression was conducted to control confounders, being of female gender was the only significant predictor of scoring 50 % and above in the knowledge test. This could be explained that female pharmacists were more concerned with breast cancer and could have acquired more knowledge compare to their male peers. Findings of this study might highlight the need to improve knowledge of male pharmacists with regard to breast cancer.

In general, pharmacists in this study were positive with regard to breast cancer health promotion. Additionally, attitude scores positively correlated with the knowledge scores. This indicated that pharmacists who were more knowledgeable of breast cancer expressed more positive attitudes toward engagement in breast cancer health promotion. Findings of this study were consistent with those reported among pharmacists in Malaysia, Qatar, and Jordan [40, 42, 48]. Community pharmacists are one of the most easily accessible and trusted healthcare providers. The professional roles of pharmacists have witnessed steady expansion and pharmacists are increasingly becoming engaged in health promotion of different diseases [63, 64]. Recent studies have highlighted the possibility of expanding the roles of pharmacists in breast cancer health promotion [40–42, 48]. Findings of this study highlighted interest among community pharmacists in Palestine to engage in breast cancer promotion.

Findings of this study indicated that lack of reimbursement, lack of enough personnel, lack of time, and fear of offending patients limited engagement of the community pharmacists in breast cancer health promotion. Our findings were consistent with those previously reported among community pharmacists elsewhere [40, 42, 48]. Probably, decision makers have to address these barriers to increase engagement of pharmacists in breast cancer health promotion [27, 62]. Findings of this study might be generalized with caution to all community pharmacists in Palestine and probably those with similar characteristics in approximately similar healthcare settings.

### Strengths and limitations

The results of this study might be interpreted considering a number of strengths and limitations. This study was the first to be conducted among community pharmacists in Palestine. Community pharmacists in Palestine are trusted and easily accessible healthcare providers who could be used in promoting the health of patients with breast cancer. Second, the questionnaire used in this study was previously used in different settings elsewhere. However, adequate pilot testing was used in this study to re-validate the questionnaire before it was used. Third, pharmacists’ knowledge, attitudes, willingness to engage, confidence, and barriers to engagement in health promotion of breast cancer were investigated in this study. This multidimensional study might provide broad information to decision makers who wish to engage community pharmacists in breast cancer health promotion.

The limitations associated with this study include the following. First, 0.5 point was deducted for each knowledge wrong answer. Scores could have been different in case we did not deduct points as penalty for each wrong answer. Second, attitudes and beliefs collected in this study are self-reported. Third, opinions of patients and decision makers were not collected in this study. Future studies should include views and opinions of patients with breast cancer and decision makers in health authorities in Palestine.

## Conclusions

This study shed light on the role of community pharmacists in breast cancer health promotion. Pharmacists had good knowledge of breast cancer and positive attitudes toward promoting the health of patients with breast cancer. Further studies are still needed to determine how to integrate community pharmacists in the team of healthcare providers caring for patients with breast cancer.

## Supplementary Information

**Additional file 1**

## Data Availability

The datasets used and/or analyzed during the current study are available from the corresponding author on reasonable request.

## Abbreviations

STROBE: Reporting of Observational Studies in Epidemiology
BSc: Bachelor of Science
CI: Confidence interval
IQR: Interquartile range
IRB: Institutional Review Board
MSc: Master of Science
Pharm.D: Doctor of Pharmacy
PhD: Doctor of Philosophy
χ^2^: Chi-square

## Declarations

### **Authors’ Information**

Ramzi Shawahna, Associate Professor, Department of Physiology, Pharmacology and Toxicology, Faculty of Medicine and Health Sciences, An-Najah National University, Nablus, Palestine, An-Najah BioSciences Unit, Centre for Poisons Control, Chemical and Biological Analyses, An-Najah National University, Nablus, Palestine. Hiba Awawdeh is a Master of Clinical Pharmacy candidate, Faculty of Graduate Studies, An-Najah National University, Nablus, Palestine.
