# Supplementary material for: Pharmacists’ knowledge, attitudes, beliefs, and barriers toward breast cancer health promotion: a cross-sectional study in the Palestinian territories
Source: BMC Health Serv Res. 2021 May 6;21:429. doi: 10.1186/s12913-021-06458-5 (PMC8101222; doi:10.1186/s12913-021-06458-5)
Supplement: Supplementary file 1 — Additional file 1 [file 12913_2021_6458_MOESM1_ESM.docx]

**Supplementary materials for the manuscript:**

**Pharmacists’ knowledge, attitudes, beliefs, and barriers toward** **breast cancer health promotion: a cross-sectional study in the Palestinian territories**

Ramzi Shawahna^1,2*^, Hiba Awawdeh^3^

^1^Department of Physiology, Pharmacology and Toxicology, Faculty of Medicine and Health Sciences, An-Najah National University, Nablus, Palestine

^2^An-Najah BioSciences Unit, Centre for Poisons Control, Chemical and Biological Analyses, An-Najah National University, Nablus, Palestine

^3^Faculty of Graduate Studies, An-Najah National University, Nablus, Palestine

**^*^Correspondence:**

Ramzi Shawahna, PhD, Department of Physiology, Pharmacology and Toxicology, Faculty of Medicine & Health Sciences, New Campus, Building: 19, Office: 1340, An-Najah National University, P.O. Box 7, Nablus, Palestine

Phone: + (970) 923 45113 ext 2772

Phone: + (970) 92349739

Email: [ramzi_shawahna@hotmail.com](mailto:ramzi_shawahna@hotmail.com)

**Supplementary File 1**

Adherence to the Reporting of Observational Studies in Epidemiology (STROBE) Statement

|  | Item No | Recommendation | Page No |
| --- | --- | --- | --- |
| **Title and abstract** | 1 | (*a*) Indicate the study’s design with a commonly used term in the title or the abstract | Please see the title and the abstract |
|  |  | (*b*) Provide in the abstract an informative and balanced summary of what was done and what was found | Please see the abstract |
| Introduction | | | |
| Background/rationale | 2 | Explain the scientific background and rationale for the investigation being reported | Introduction: Page 3, 3^rd^ and 4^th^ paragraphs |
| Objectives | 3 | State specific objectives, including any prespecified hypotheses | Introduction: Page 4, last paragraph |
| Methods | | | |
| Study design | 4 | Present key elements of study design early in the paper | Methods, Section: Study design |
| Setting | 5 | Describe the setting, locations, and relevant dates, including periods of recruitment, exposure, follow-up, and data collection | Methods, Section: Population, sample size, recruitment, inclusion, and exclusion criteria |
| Participants | 6 | (*a*) Give the eligibility criteria, and the sources and methods of selection of participants | Methods, Section: Population, sample size, recruitment, inclusion, and exclusion criteria |
| Variables | 7 | Clearly define all outcomes, exposures, predictors, potential confounders, and effect modifiers. Give diagnostic criteria, if applicable | Methods, Section: The study tool |
| Data sources/ measurement | 8* | For each variable of interest, give sources of data and details of methods of assessment (measurement). Describe comparability of assessment methods if there is more than one group | Methods, Section: The study tool |
| Bias | 9 | Describe any efforts to address potential sources of bias | Methods, Section: Pilot testing, stability of scores, and internal consistency of the study tool |
| Study size | 10 | Explain how the study size was arrived at | Methods, Section: Population, sample size, recruitment, inclusion, and exclusion criteria |
| Quantitative variables | 11 | Explain how quantitative variables were handled in the analyses. If applicable, describe which groupings were chosen and why | Methods, Section: Statistical analysis |
| Statistical methods | 12 | (*a*) Describe all statistical methods, including those used to control for confounding | Methods, Section: Statistical analysis |
|  |  | (*b*) Describe any methods used to examine subgroups and interactions | Methods, Section: Statistical analysis |
|  |  | (*c*) Explain how missing data were addressed | n/a |
|  |  | (*d*) If applicable, describe analytical methods taking account of sampling strategy | Methods, Section: Statistical analysis |
|  |  | (*e*) Describe any sensitivity analyses | n/a |
| Results | | | |
| Participants | 13* | (a) Report numbers of individuals at each stage of study—eg numbers potentially eligible, examined for eligibility, confirmed eligible, included in the study, completing follow-up, and analysed | Results, Section: The community pharmacists who took part in the study and Table 1 |
|  |  | (b) Give reasons for non-participation at each stage | Results, Section: The community pharmacists who took part in the study |
|  |  | (c) Consider use of a flow diagram | n/a |
| Descriptive data | 14* | (a) Give characteristics of study participants (eg demographic, clinical, social) and information on exposures and potential confounders | Results, Section: The community pharmacists who took part in the study and Table 1 |
|  |  | (b) Indicate number of participants with missing data for each variable of interest | n/a |
| Outcome data | 15* | Report numbers of outcome events or summary measures | Results, Section: The community pharmacists who took part in the study and Table 1 |
| Main results | 16 | (*a*) Give unadjusted estimates and, if applicable, confounder-adjusted estimates and their precision (eg, 95% confidence interval). Make clear which confounders were adjusted for and why they were included | Tables 1-6 |
|  |  | (*b*) Report category boundaries when continuous variables were categorized | Tables 1-6 |
|  |  | (*c*) If relevant, consider translating estimates of relative risk into absolute risk for a meaningful time period | Tables 1-6 |
| Other analyses | 17 | Report other analyses done—eg analyses of subgroups and interactions, and sensitivity analyses | Tables 1-6 |
| Discussion | | | |
| Key results | 18 | Summarise key results with reference to study objectives | Discussion, 1^st^ paragraph |
| Limitations | 19 | Discuss limitations of the study, taking into account sources of potential bias or imprecision. Discuss both direction and magnitude of any potential bias | Discussion, Section: Strengths and limitations |
| Interpretation | 20 | Give a cautious overall interpretation of results considering objectives, limitations, multiplicity of analyses, results from similar studies, and other relevant evidence | Discussion, Paragraphs 2-4 |
| Generalisability | 21 | Discuss the generalisability (external validity) of the study results | Discussion, Paragraph 4. |
| Other information | | | |
| Funding | 22 | Give the source of funding and the role of the funders for the present study and, if applicable, for the original study on which the present article is based | Declarations |

**Supplementary File 2**

Distribution of community pharmacies in the governorates of the West Bank of Palestine
